# Supplementary figures and images for: SAD-1 kinase controls presynaptic phase separation by relieving SYD-2/Liprin-α autoinhibition
Source: PLoS Biol. 2023 Dec 4;21(12):e3002421. doi: 10.1371/journal.pbio.3002421 (PMC10695385; doi:10.1371/journal.pbio.3002421)

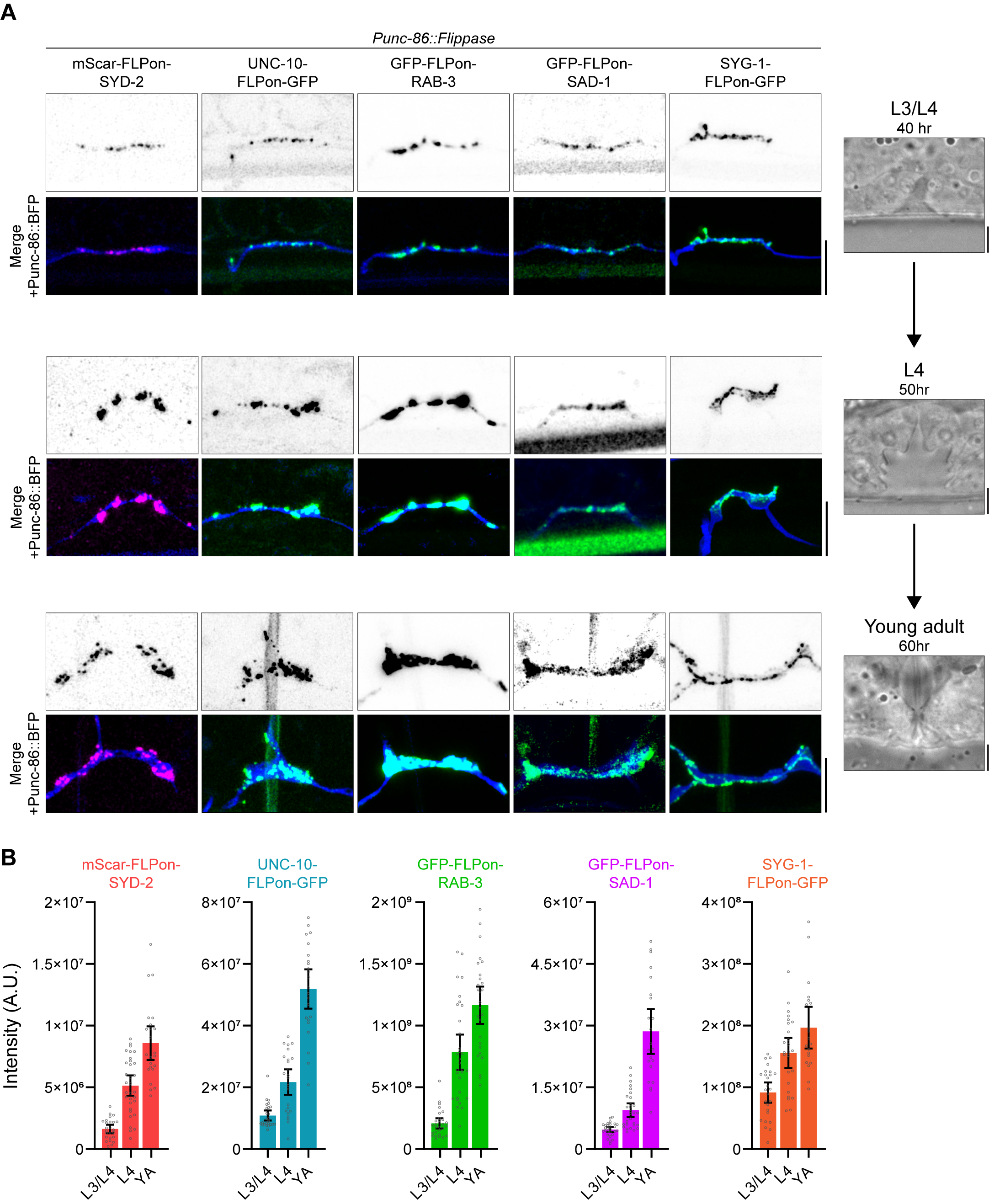

Supplement: S1 Fig — (A) Representative Airyscan superresolution images of the indicated endogenously tagged synaptic proteins at 3 developmental time points. Brightfield images show vulval morphology used to identify each time point. Hours indicate time elapsed since starved and synchronized L1 animals were reintroduced to food at 20°C. (B) Quantification of HSN synapse intensity of each marker from (A). Underlying data is available in S1 Data. (TIF) [file pbio.3002421.s001.tif]

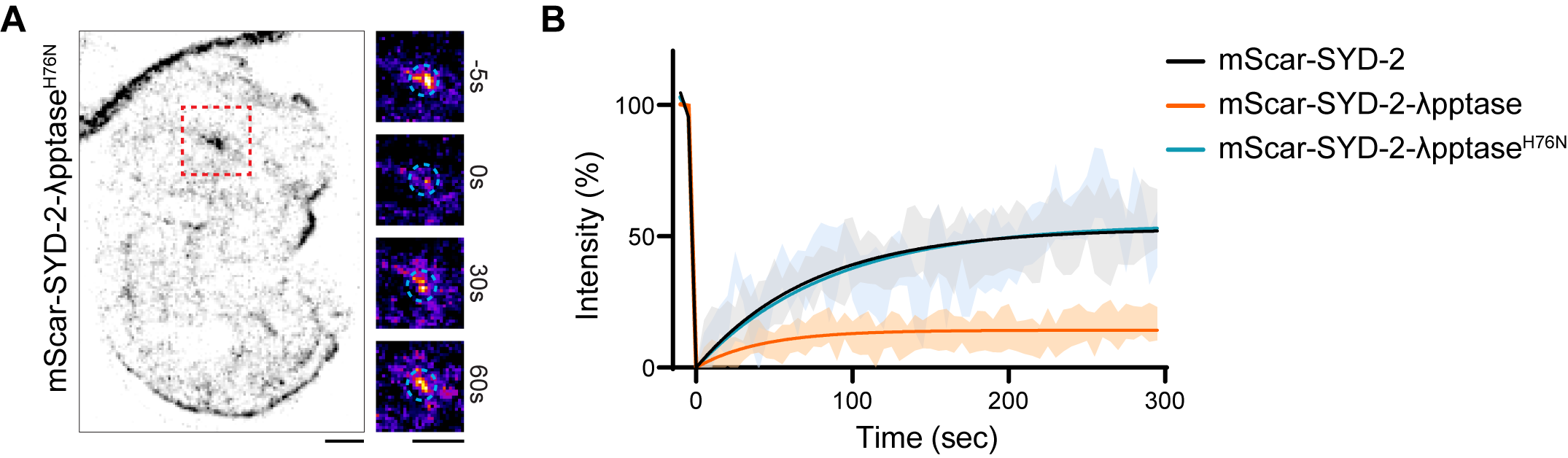

Supplement: S2 Fig — (A) Fluorescence recovery after photobleaching of endogenous mScarlet-SYD-2-λpptaseH76N catalytically inactive control at embryonic nerve ring synapses to measure dynamics. Scale bars, 5 μm. (B) Quantification of FRAP in (A). Wild-type and λpptase data included from Fig 1C. Underlying data is available in S1 Data. (TIF) [file pbio.3002421.s002.tif]

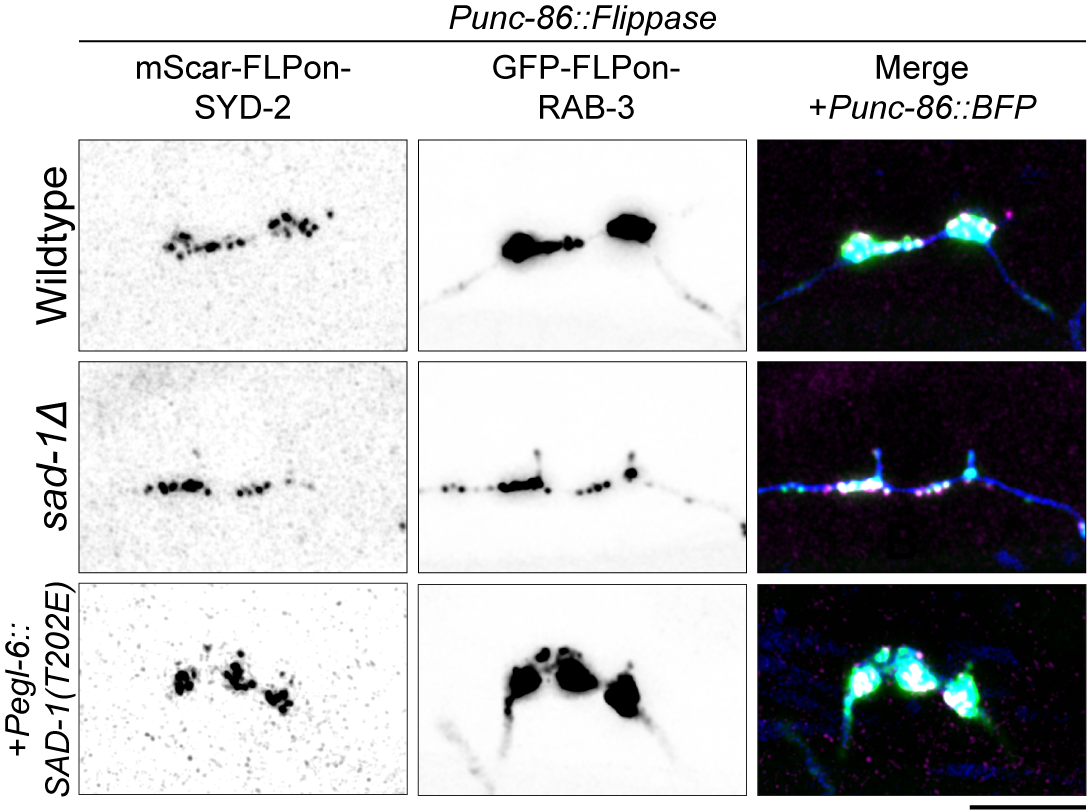

Supplement: S3 Fig — Synapse formation phenotypes visualized with Airyscan superresolution imaging of endogenous GFP-RAB-3 in the indicated mutants. Scale bars, 5 μm. (TIF) [file pbio.3002421.s003.tif]

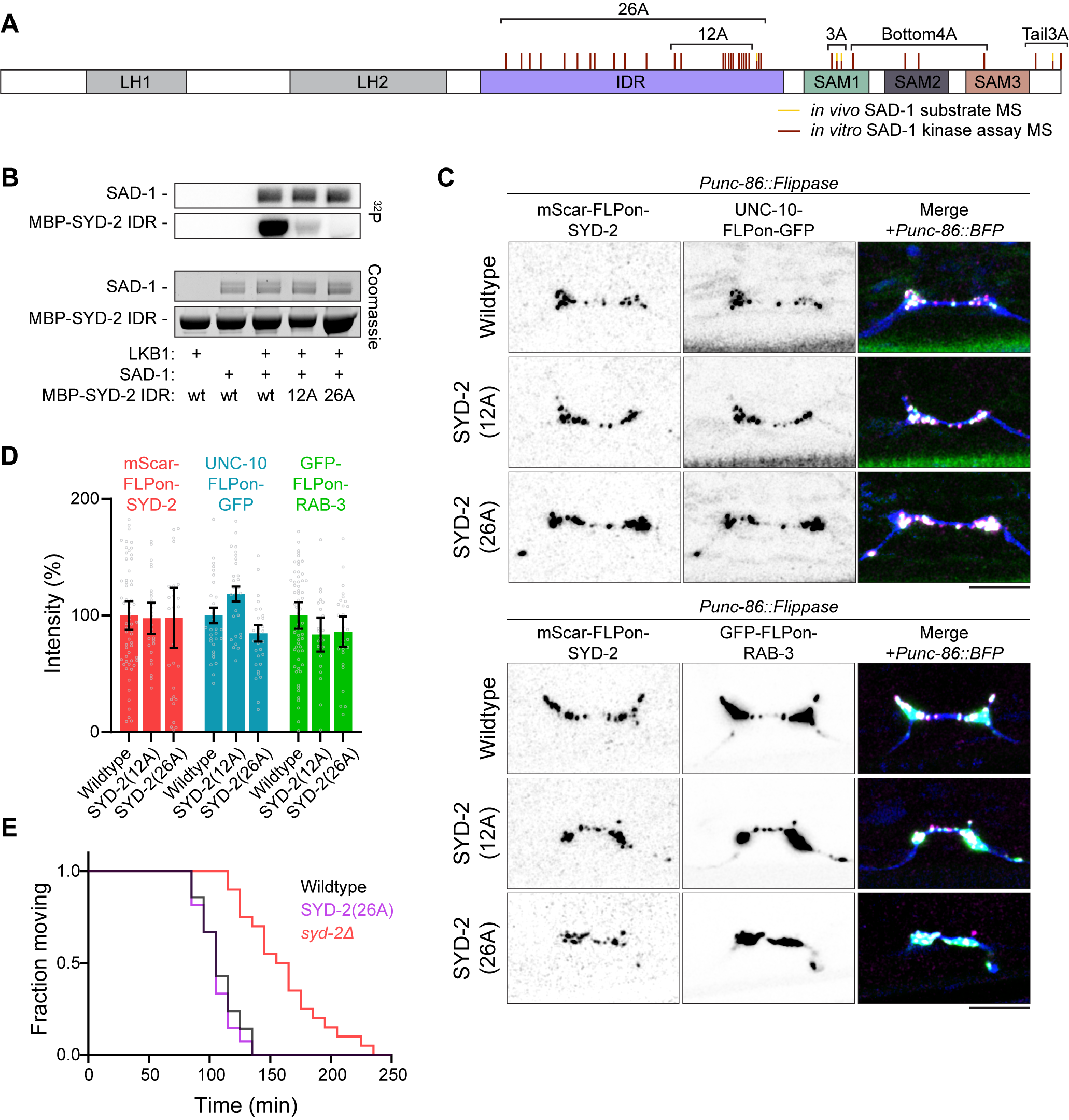

Supplement: S4 Fig — (A) SYD-2 phosphosites identified from in vivo phosphoproteomics (yellow) or in vitro kinase assays (burgundy). Sites were grouped based on location for subsequent testing. See S1 and S2 Tables. (B) In vitro kinase assay between SAD-1 and SYD-2 IDR with or without phosphosite mutations. SAD-1 is activated by the LKB-1 kinase complex. (C) HSN synapse formation phenotypes visualized with Airyscan superresolution imaging of endogenous fluorescent tags in the indicated mutants. Scale bars, 5 μm. (D) Quantification of HSN intensities in (C). No significant difference in synapse formation was seen in IDR phosphomutants. (E) Aldicarb synaptic transmission assay shows no defects in an SYD-2 IDR phosphomutant. Extended time to paralysis on 1 mM Aldicarb indicates defective synaptic transmission. n > 20 for each genotype. Underlying data is available in S1 Data. (TIF) [file pbio.3002421.s004.tif]

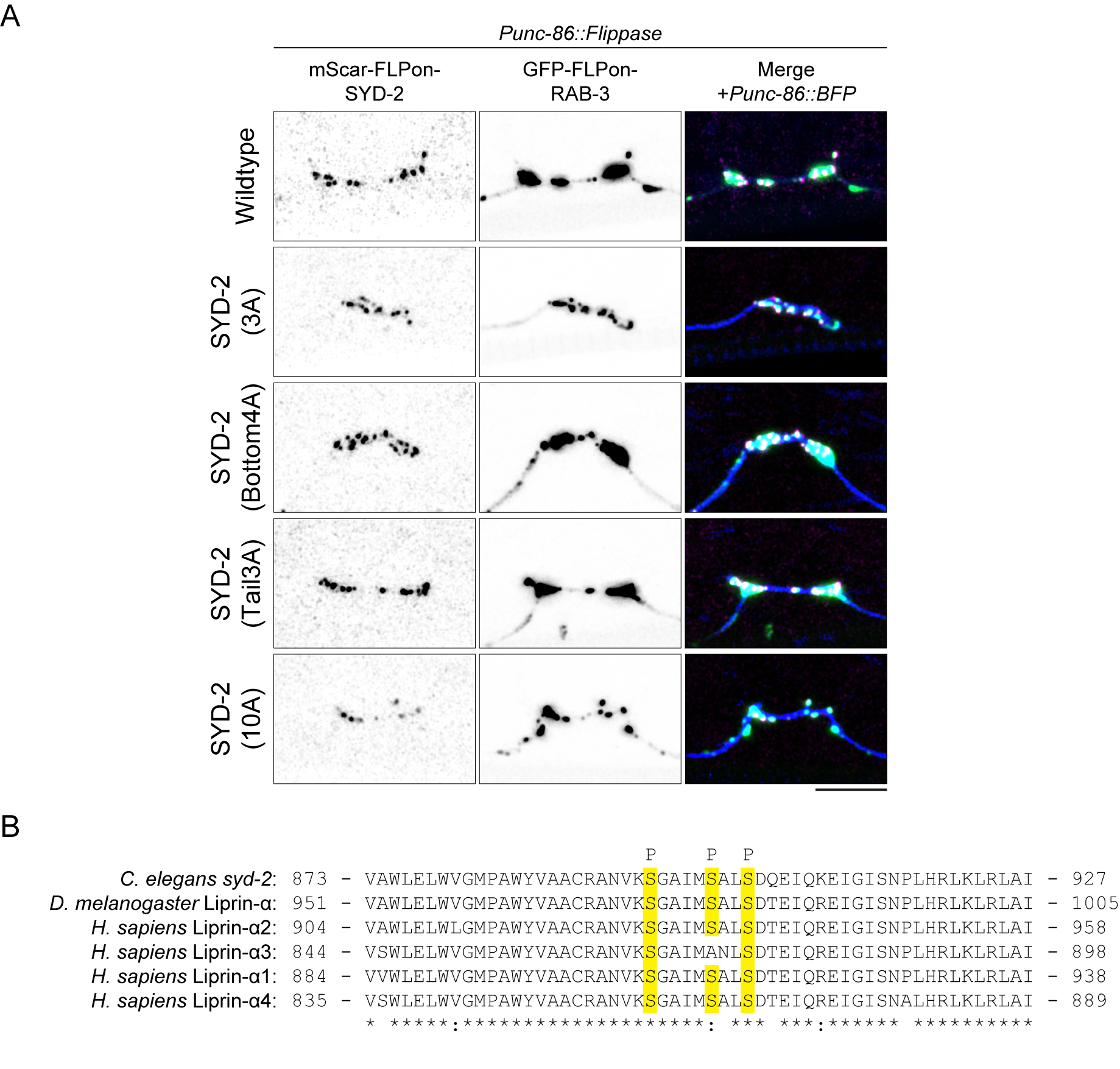

Supplement: S5 Fig — (A) HSN synapse formation phenotypes visualized with Airyscan superresolution imaging of endogenous GFP-RAB-3 in the indicated mutants. Scale bars, 5 μm. Quantification is presented in Fig 4B. (B) Alignment of C. elegans, D. melanogaster, and H. sapiens SYD-2/Liprin-αs showing conservation of 3 SAM phosphosites. (TIF) [file pbio.3002421.s005.tif]

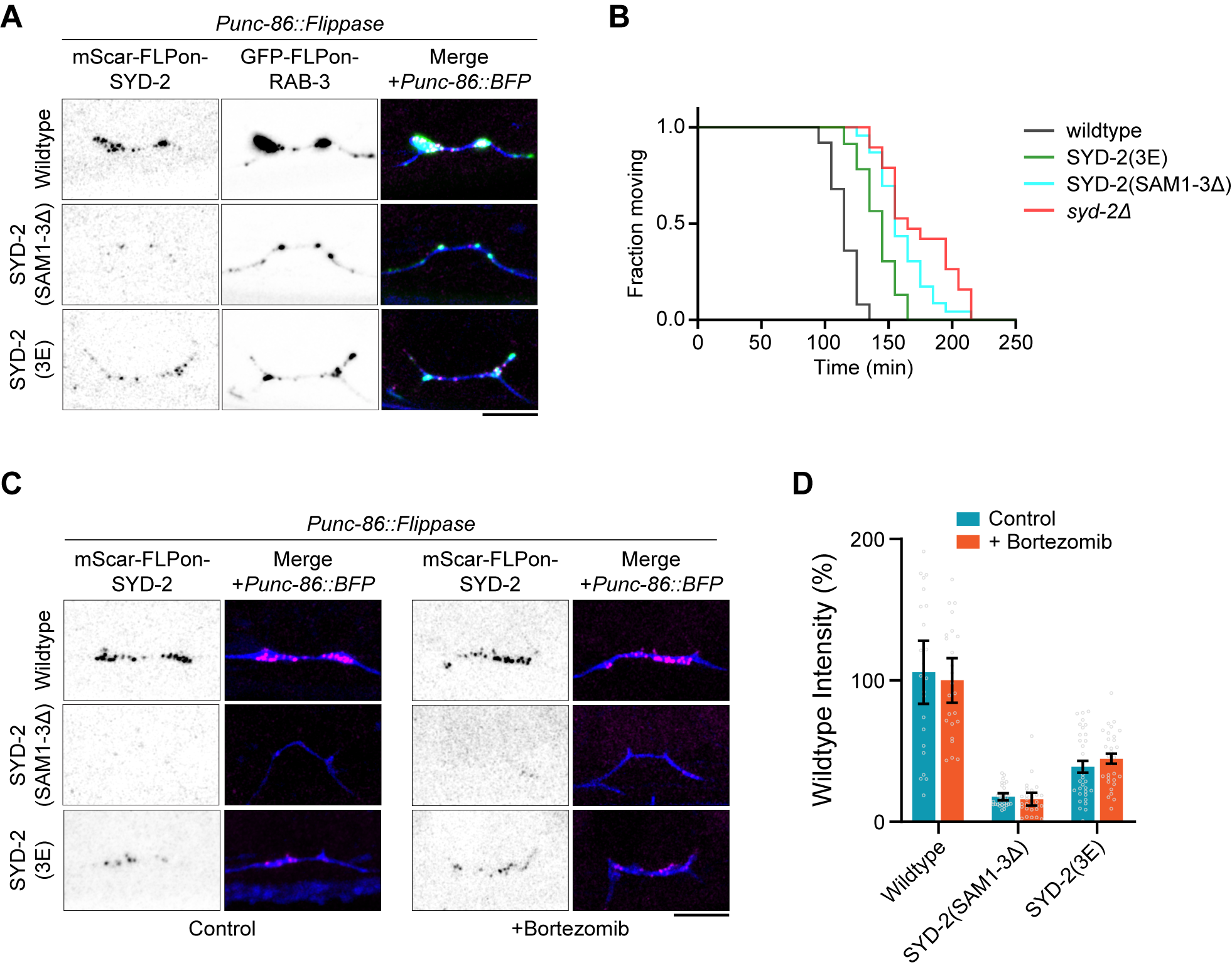

Supplement: S6 Fig — (A) HSN synapse formation phenotypes visualized with Airyscan superresolution imaging of endogenous fluorescent tags in the indicated mutants. Removal of SAM domains or mimicking SAD-1 phosphorylation leads to reduced SYD-2 at HSN synapses. See quantification in Fig 6B. Scale bar, 5 μm. (B) Aldicarb synaptic transmission assay. Extended time to paralysis on 1 mM Aldicarb indicates defective synaptic transmission. n > 20 for each genotype. (C) HSN synapse formation phenotypes visualized with Airyscan superresolution imaging of endogenous fluorescent tags in the indicated mutants; 10 μm of the proteasome inhibitor bortezomib or DMSO as a control was added to the indicated animals for 4 h prior to imaging. Scale bar, 5 μm. (D) Quantification of SYD-2 synaptic intensities in (C). Underlying data is available in S1 Data. (TIF) [file pbio.3002421.s006.tif]

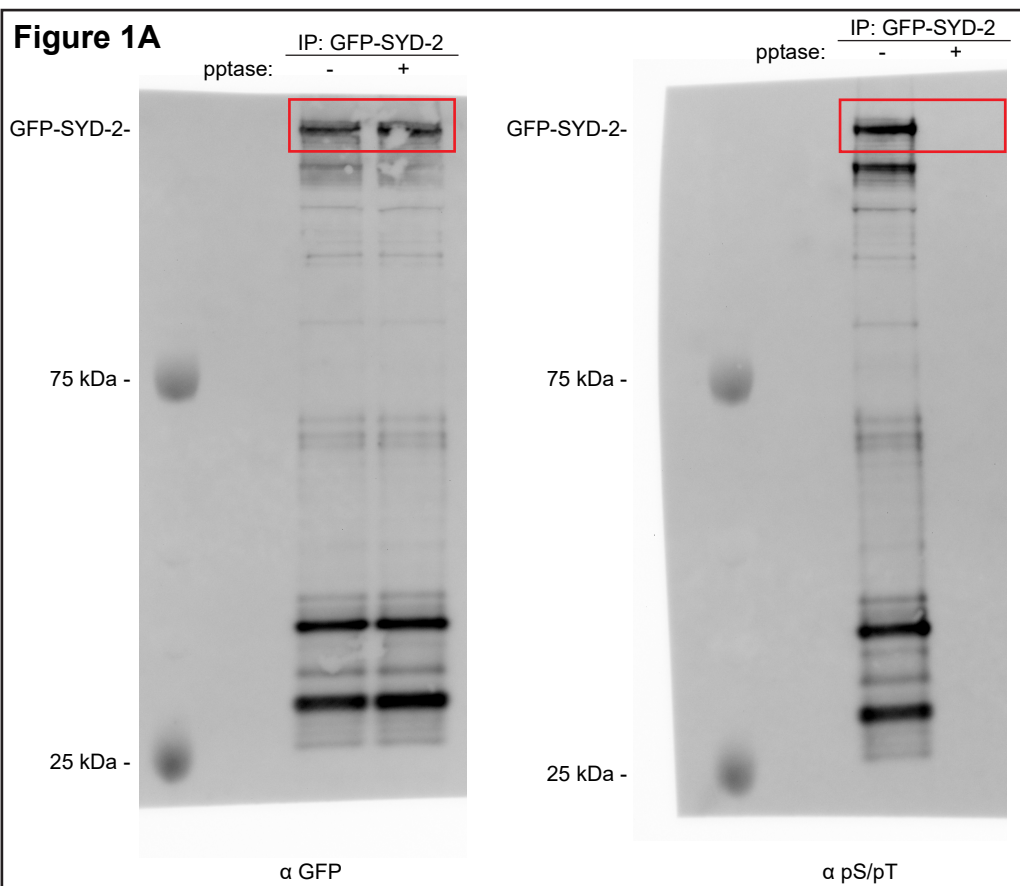

**Figure 1B**

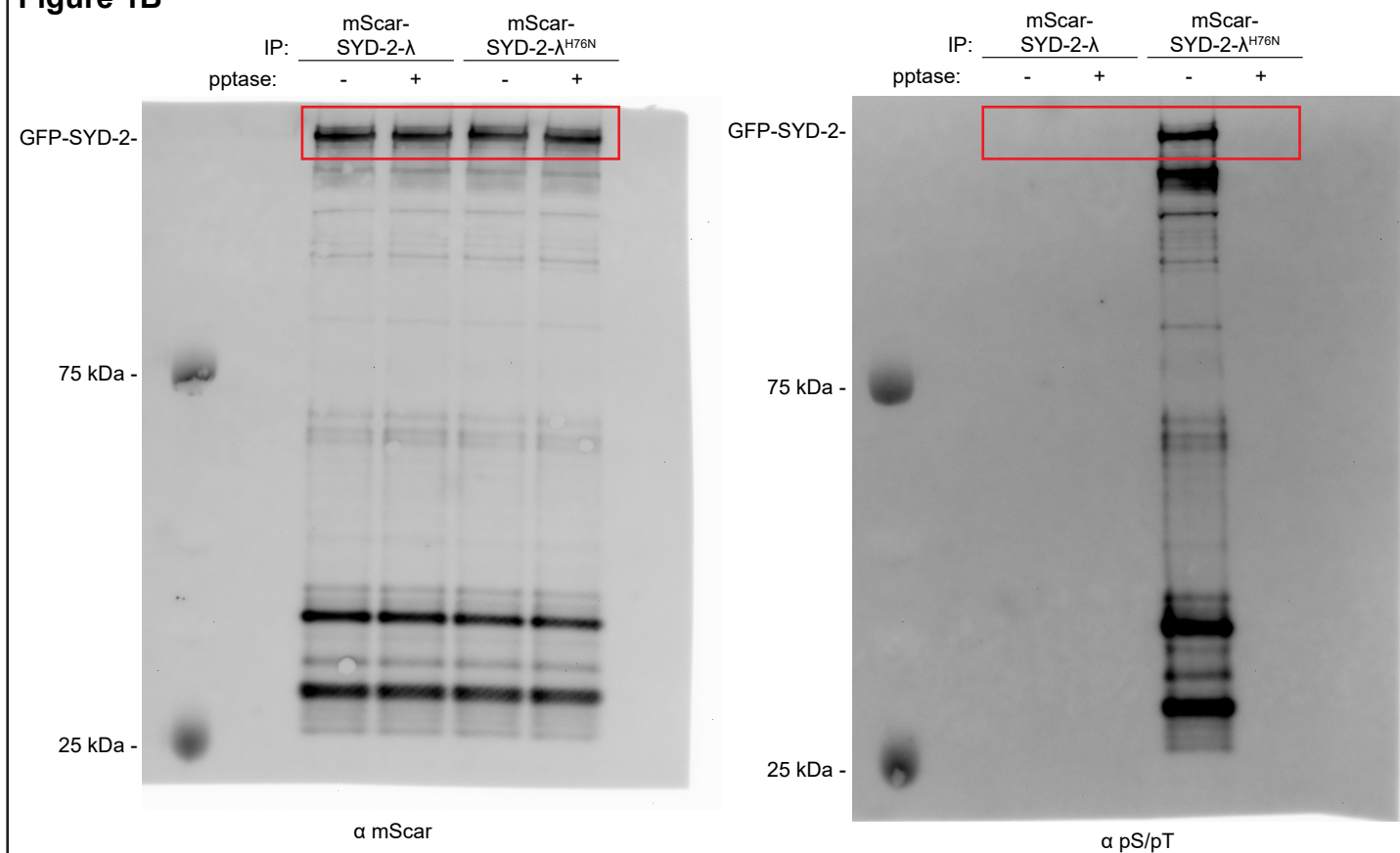

Figure 3B & 3D

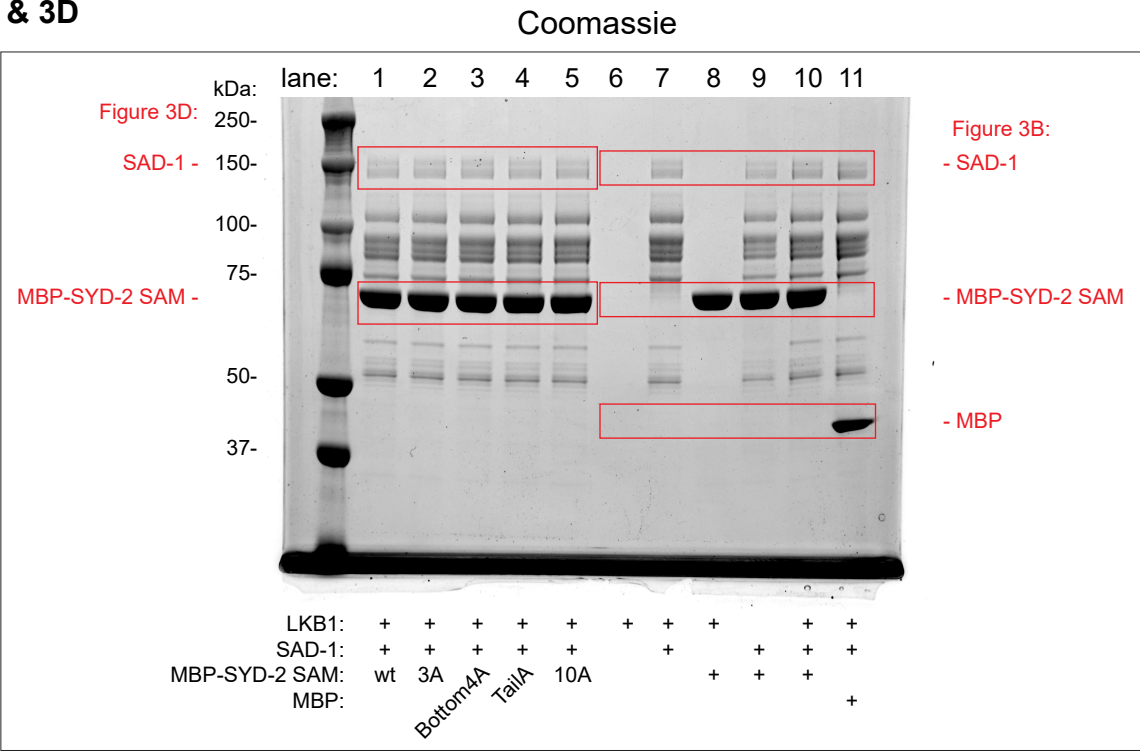

<sup>32</sup>P low contrast

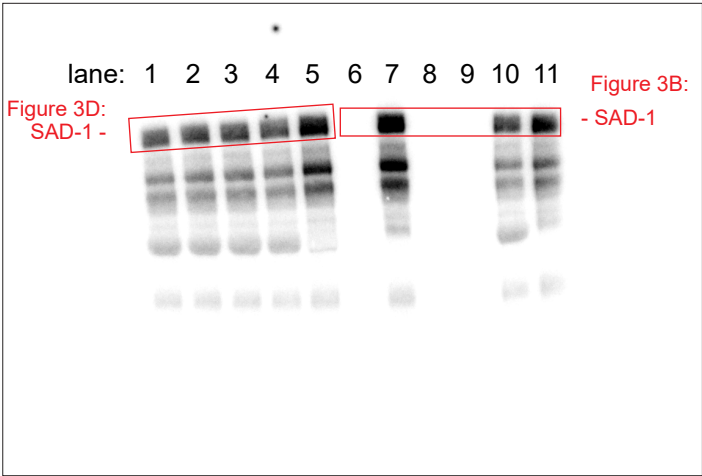

<sup>32</sup>P high contrast

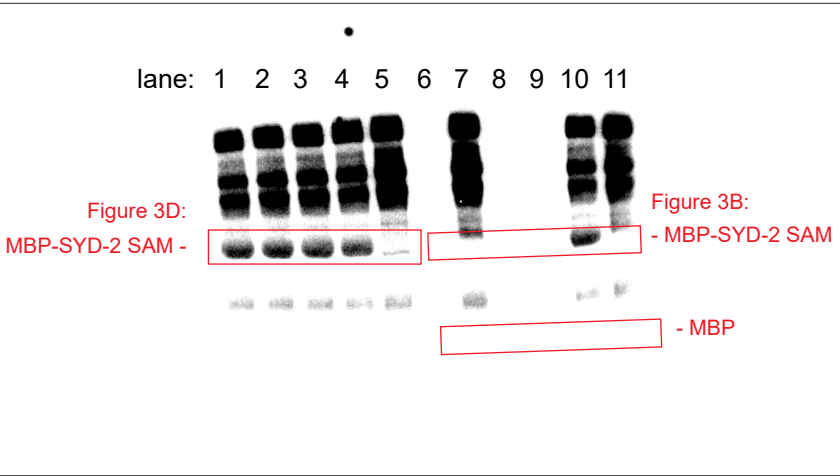

**Figure 7**

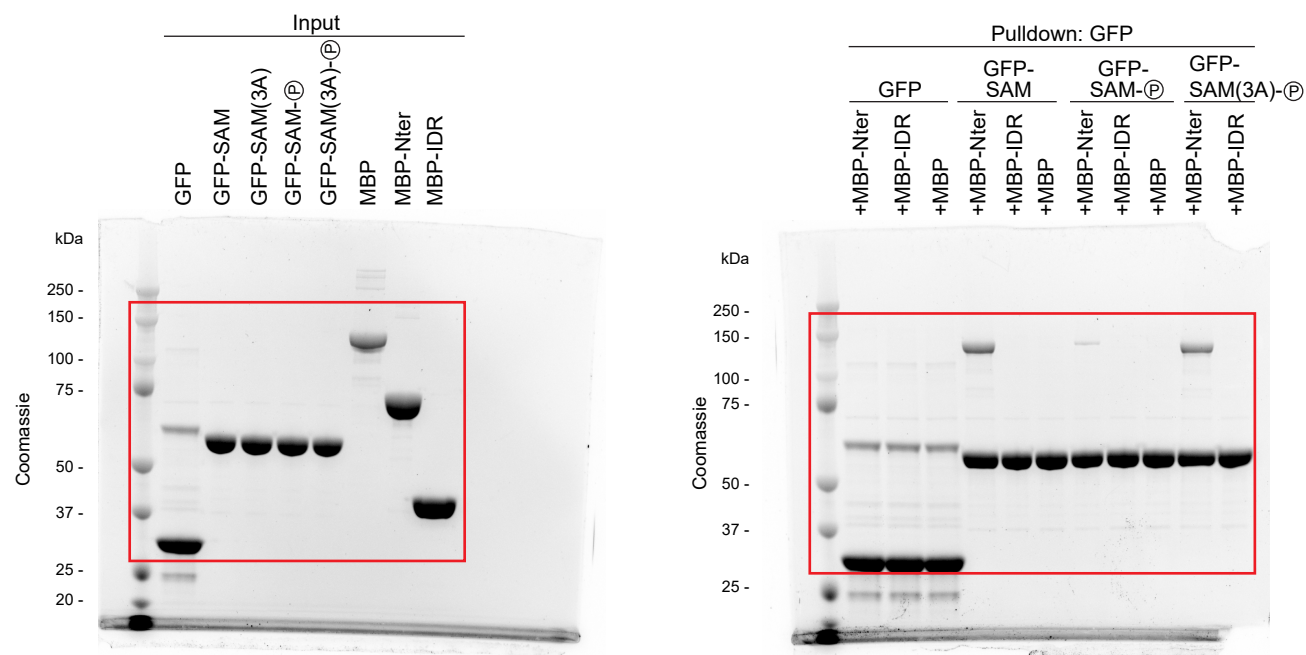

Figure S4B

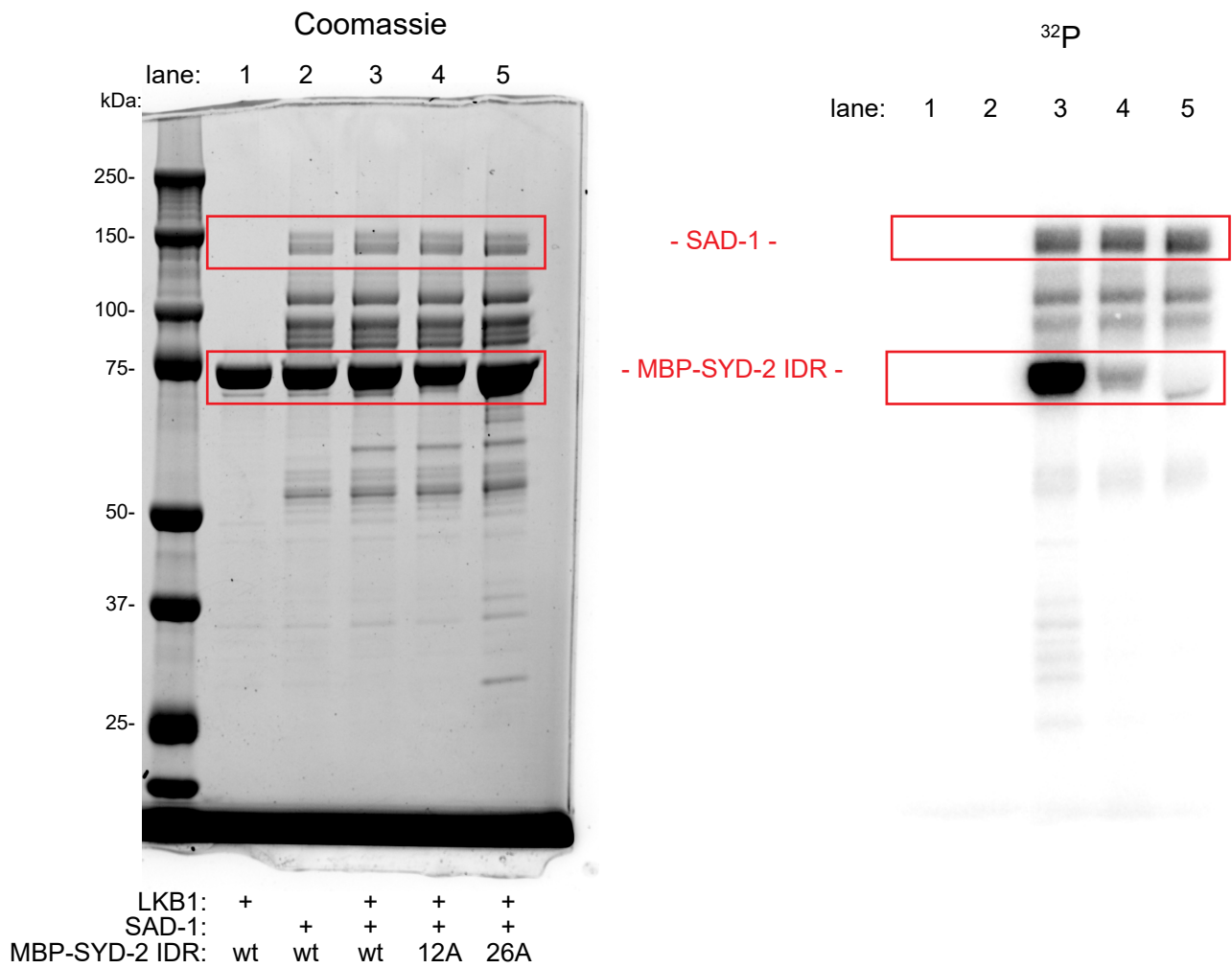

Supplement: S1 Raw Images — (PDF) [file pbio.3002421.s012.pdf]
