# Supplementary material for: SAD-1 kinase controls presynaptic phase separation by relieving SYD-2/Liprin-α autoinhibition
Source: PLoS Biol. 2023 Dec 4;21(12):e3002421. doi: 10.1371/journal.pbio.3002421 (PMC10695385; doi:10.1371/journal.pbio.3002421)
Supplement: Attachment — Submitted filename: Response to Reviewers_v2.docx [file pbio.3002421.s013.docx]

**Response to Reviewers**

Reviewer #1 (Stephan Sigrist, signs review): Every presynapse builds a core active zone scaffold, where ion channels cluster and synaptic vesicles release their neurotransmitters. The protein composition of active zones (AZs) is rather well characterized, with Syd-2 aka Liprin, ELKS/BRP and RIM-BP proteins being conserved core scaffold components. Recent, prominently published work by the Shen lab has shown that Syd-2/ELKS "scaffold" adopts a liquid character in association with driving developmental AZ assembly, but later matures into a more solid structure. Regulatory principles confining such liquid-solid phase transitions to properly steer active zone assembly so far remain largely enigmatic, however.

Here, using a broad spectrum of genetic and biochemical methods the Shen lab investigates the role of the Sad-1 kinase in the control of Syd-2 liquidity and thus AZ scaffold assembly. They in my eyes provide convincing evidence for Sad-1 phosphorylation of Syd-2 at/within the first Syd-2 SAM-domain controls AZ assembly in conjunction with controlling liquidity.

This study in my eyes provides important evidence to exemplify Syd-2 phosphorylation dependent recruitment of "downstream components" such as Rim/Unc10 and Rab3. They convincingly show that SYD-2's SAM domains directly interact with its N-terminus to inhibit phase separation, which is released by specific SAD-1 phosphorylation in the first SAM domain.

This becomes possible by a real tour de force using an impressive array of on-locus point mutations in syd-2. I do recommend publication of this study in Plos Biology essentially as is.

Comments: As said, I am very positive and suggest publication. This said, the object of study, though timely and relevant, is intrinsically difficult and drawing undisputably causal relations still challenging (though they are getting close by doing domain swops). In my eyes, extrapolating local viscosity/liquidity within AZs by "global" FRAP is a somewhat coarse approach, which might mask different AZ subzones and subpopulations of the relevant proteins. This reviewer would appreciate to see this somewhat more reflected in the discussion of their results.

We thank Reviewer 1 for the assessment of the significance of this work. We agree that FRAP is a coarse approach. It remains, however, one of the few ways to directly interrogate phase separation dynamics *in vivo*. Because of the small size of synapses, we are limited to bleaching entire active zones; therefore, different subpopulations within an active zone would certainly be masked by this technique. We have attempted to use more advanced techniques such as fluorescence cross-correlation spectroscopy but have still been hampered by resolution and diffraction limits to answer questions such as this.

We have expanded our explanation of these FRAP assays in the results section to better reflect these considerations (lines 86-90).

Additional points:

- Do the in vitro and in vivo identified Sad-1 sites are in line with a certain consensus? Following this, are the putatively artefactual IDR phospho-sites less close to a consensus? Concerning their homology considerations, I do suggest to also mention the Drosophila situation concerning the relevant Sad-1 sites, as Drosophila also is a highly relevant model in AZ research.

We have not been able to discern a consensus sequence for SAD-1 based on the identified *in vivo* phosphosites, and one has not been reported previously. It is likely that the disordered IDR is an easy substrate for SAD-1 *in vitro* and these sites do not reflect *in vivo* consensus.

We have added a new figure showing that the phosphosites with human and Drosophila Liprin-αs (Figure S5B) are conserved and added Drosophila Liprin-α to our discussion (lines 172-3 and 274-6).

- They say: "Wildtype SYD-2 at nascent synapses recovers quickly after photobleaching, due to liquid condensate formation and rapid exchange between condensate and cytoplasmic pools". How do they know that there is exchange with cytoplasmic pools? Couldn't it be all exchange between AZs? I am also rather confident that extra-AZ pools do exist, but would appreciate to hear their arguments in this point.

The FRAP assay is intended to simply measure the exchange between an AZ SYD-2 condensate and any additional pool, in order to correlate these dynamics with phase separation. It is challenging to determine exactly where the photobleaching recovery occurs from. However, in the FRAP time courses, we do not observe movement of neighboring AZs that would contribute to recovery. Therefore, we favor the view that exchange is occurring with a cytoplasmic pool. The cytoplasmic pool likely also exchanges with neighboring AZs, and we have modified the text to reflect this possibility (lines 89-90).

- What exactly are the arguments that it is the IDR entertaining dominating critical interactions to other active zone proteins? I thought the more N-terminal coiled coil domains of Syd-2/Liprin might be relevant (as well)?

We do not argue that the IDR is involved in interactions with other AZ proteins. Interactions have been mapped to N-terminal coiled-coils and C-terminal SAM domains surrounding the IDR. The function of the IDR appears to be phase separation alone so far. In fact, we can completely replace SYD-2’s IDR with that from the unrelated protein FUS and retain function (McDonald et al. 2020).

- Again concerning discussion: do they think of the AZ liquidity to be reversible? Interestingly, the Nter + SAM droplets formed at high concentration were not dynamic but resembled aggregates rather than liquid condensates. Thus: is the transport of Syd-2 really operating in a closed form, not associated with other AZ proteins?

We have not observed re-liquification of AZs, but the possibility is intriguing. FRAP of synapses in the adult nerve ring showed little recovery on the timescale of minutes to hours (McDonald et al. 2020).

We have not investigated transport in this study but from previous studies consider it likely that SYD-2 transport occurs in the “closed”, soluble form. We previously imaged synapse formation in detail and directly observed AZ assembly out of a diffuse axonal pool (Lipton et al. (2018) *Cell Rep*, Figure 3 and S3) with limited visible transport to nascent synapses. It is possible that some SYD-2 scaffolding interactions occur before or during transport, but we have not observed large transport complexes.

- Which states do the differentiate: liquid condensate to recruit, "aggregate" state once recruitment is ended and AZs are matured?

Our current and previous data suggest: 1) a soluble state in the cytoplasm and axon, 2) a developing active zone liquid condensate state, and 3) a solid condensate state in mature synapses.

-Do AZ scaffolds in Delta-SAM mutants loose liquidity prematurely? Are there extra-AZ axonal/cell body aggregates forming for any of their mutants? Especially the phosphomimetic mutants which should suffer "premature liquidity"?

We had also considered this idea and hypothesized that the SAMΔ or 3E mutants would result in precocious phase separation due to the loss of inhibition. However, we observed that these mutants had drastically decreased levels at synapses.

We have performed additional experiments that clarify this phenotype. We reasoned that if SAMΔ and 3E mutants were precociously phase separating but not reaching synapses, this may be occurring in the cell body immediately post-translation. We imaged SYD-2 in HSN cell bodies and found a low level of diffuse, soluble protein with occasional puncta, and no differences between wildtype and SAMΔ or 3E mutants. We further considered the possibility that precociously phase-separating SYD-2 would be degraded. To test this, we treated animals with a proteasome inhibitor before imaging SYD-2 in cell bodies. Here, we found an increased amount of SYD-2(SAMΔ) and SYD-2(3E) in puncta compared to wildtype. We conclude that these mutants are normally degraded, likely due to precocious phase separation, resulting in low synaptic levels. When proteasomal degradation is blocked, they accumulate in cell body puncta. We present this data in a new Figure 6 and lines 186-203.

- How do they think concerning ELKS, major subject of investigation in their previous LLPS study: transporting independent of Syd-2? Also in an aggregated form? SRPK might keep ELKS/BRP in a liquid transport form in Drosophila, do they think ELKS/RimBP complexes might transport differently from Syd-2?

Phosphorylation by SRPK is an intriguing hypothesis for control of ELKS’ phase separation considering the Drosophila BRP data. We have not investigated the transport of SYD-2 or ELKS in this manuscript, but our previous studies have indicated assembly of ELKS at AZs occurs out of a diffuse axonal pool (Lipton et al. (2018) *Cell Rep*), perhaps suggesting soluble transport. The *C. elegans* homolog of SRPK is a lethal gene. A conditional allele could be generated but that will take some time.

- "A complete 10A mutant reproduced the synapse formation defects of the 3A mutant" it is somewhat hard to keep track with all their genotype nomenclature, maybe a little more of in between explanation/reiteration might be helpful here.

We have included additional reiteration of these mutants in the text (lines 162-173).

Reviewer #2: In this manuscript, McDonald et al present their interesting and intriguing findings on the role SAD-1 kinase in regulating SYD-2 and presynaptic assembly in C. elegans. They used a combination of genetic, biochemical, and imaging approaches to show that SAD-1 phosphorylates SYD-2 on three serine residues in the first SAM domain, which relieves an autoinhibitory interaction between the N-terminal coiled coils and the C-terminal SAM domains. The authors also showed that SAD-1 is localized to nascent synapses by upstream synaptic cell adhesion molecules. Their data provides evidence for the novel regulation of SYD-2 activation by a presynaptic kinase. Considering the high conservation of the three phosphorylation sites in the liprin-alpha family, it is plausible that the phosphorylation-mediated activity regulation may extend to mammalian liprin-alpha proteins. Thus, this study is important. However, the proposed phase separation-based mechanism lacks robust support from their data, and an alternative explanation involving the regulation of specific protein-protein interactions could also be considered. I recommend the authors to strengthen the phase separation part of the manuscript and provide additional evidence to support their claim.

Major concerns

1. The authors propose that SYD-2 phase separation is regulated by Nter-SAM interaction. However, the related data presented in Figure 6 is not convincing. The main concern lies in the low quality of the proteins used in the biochemical analysis, which raises doubts about the reliability of the results.

a. The Nter protein they purified exhibited severe degradation, appearing as several bands in SDS-PAGE (Fig. 6E). The target protein was not even the major band observed. The use of such a degraded sample for the phase separation analysis may introduce artificial outcome or biased results. It is necessary for the authors to obtain a high-quality sample of Nter, free from degradation, to ensure the accuracy and reliability of their findings.

We have obtained purer samples of Nter, SAM, and IDR fragments and repeated the phase separation assays in Figure 7B-E, with identical results.

b. The GFP-SAM protein used in the biochemical assays was also affected by partial degradation as shown in Fig. 6E. It further compromises the reliability of their interpretation of both the phase separation and pulldown results.

We have repeated the biochemical assays using purer proteins, with identical results (Figure 7E).

c. Again, in Fig. 6E, it is odd to see that all the degraded fragments of Nter were pulled down by the SAM domains. Could it be that all these degraded fragments contain SAM-binding sequence? Or, more likely, such a heavily degraded mixture nonspecifically associated with beads. The use of high-quality samples will be crucial to address this issue.

This result indeed shows that degraded/truncated fragments contain SAM binding sequences, as these bands behave similarly to the full-length species. This behavior is expected if the C-terminal IDR is degrading, with binding occurring in the N-terminal coiled-coils, as we have concluded. We did not observe any binding with MBP controls or the IDR alone, ruling out nonspecific association. Purer samples have verified these results.

d. In the presence of the SAM domains, they observed a decrease in the dynamics of Nter, as indicated by FRAP analysis (Fig. 6D). The uneven distribution of Nter protein in the condensate (Fig. 6B) also supports the alteration in protein dynamics. However, it is essential to note that a decrease in the dynamics of protein condensates does not necessarily imply a reduced ability to phase separate.

The reviewer points out important considerations with *in vitro* phase separation assays. In the context of these assays, which contain high PEG molecular crowding conditions and high protein concentrations, we have interpreted decreases in FRAP dynamics as inhibition of phase separation (consistent with our previous publication: McDonald et al. *Nature* (2020); Extended Data Fig 2A). Corroborating this interpretation, we present quantitative evidence of changes in phase separation boundaries in the phase diagrams in Figure 7C. These show that the addition of SAM domains increases the concentration and crowding required for the Nter to form droplets, hence the conclusion of an inhibition of phase separation.

e. The authors state that "binding between domains surrounding the IDR prevents its phase separation" and suggest that the release of the Nter-SAM interaction could lead to an alteration in the IDR conformation, promoting phase separation. For such an alteration to occur, it would require the surrounding domains of IDR to be present on both sides. However, the IDR was not covalently linked with the SAM domains in the experiment shown in Fig. 6B-D that was used to support the importance of Nter-SAM interaction for the inhibition of IDR-mediated phase separation. Also, considering SYD-2 exists as an oligomer mediated by the coiled coils, the Nter-SAM interaction may occur intermolecularly. In this context, the interaction could promote rather than decrease its phase separation by providing intermolecular binding valence.

The reviewer correctly points out that the *in vitro* assays were performed with unlinked Nter and SAM fragments. Yet, it is clear from our *in vitro* assays (Figure 7C-D) that SAM domains exert an inhibitory effect on Nter phase separation even when unlinked. Consistently, we find a SAM(3E) phosphomimetic partially abolishes its phase separation inhibition (Figure 7D). We can also see from the binding assay that an Nter-SAM interaction is modulated by SAM phosphorylation (Figure 7E).

Our data does not rule out additional weak interactions between SAM and phase-separating IDR domains or complex intermolecular conformations as the reviewer suggests that could influence phase separation. However, inhibitory binding between SAM and Nter is the most parsimonious mechanism to account for all of the current results.

2. The data in Fig. 6F&G is interesting but also provocative. The authors showed that the IDR of SYD-2 can be functionally replaced by FUS IDR. It can then be deduced that the SYD-2 SAM domains should also bind to FUS IDR, a highly unusual predication that needs to be verified by experiments.

Our *in vitro* binding experiments show that binding occurs between SYD-2 SAM and N-terminal coiled-coil domains, and do not require the IDR (Figure 7E). These domains remain intact in the SYD-2(IDRΔ+FUS) allele and we do not necessarily expect interaction between SAM domains and the FUS IDR. These results instead suggest N-terminal coiled-coil + SAM binding can regulate phase separation of a generic internal IDR.

3. The three important serine residues are located at the SAM1 domain, a well-characterized region known for protein-protein interactions. It raises the intriguing possibility that phosphorylation of these sites may have an impact, either positive or negative, on these interactions. Among these sites, the S896 residue is close to the liprin-beta binding surface, while S904 is located near the CASK-binding surface. The synaptic defects observed in the SAD1 deletion may be attributed to the dysregulation of SAM-mediated interactions. In the article, the authors should acknowledge such possibilities in the Discussion.

These are intriguing possibilities that we have also considered. However, it is known that SYD-2’s SAM domains are not required for synapse formation in HSN. Overexpression of a fragment containing only coiled-coils and IDR is sufficient to rescue *syd-2Δ* HSN phenotypes (Taru and Jin (2011) *J. Neurosci*).

In addition, CASK has been shown to be dispensable for HSN synapse formation (Dai et al. (2006) *Nat Neuro.*). A Liprin-β mutant in *C. elegans* has neuromuscular phenotypes, but these were found to arise from function in the muscle, not neurons (Wang et al. (2009) *Neurosci Bull*). Therefore, we consider alteration of binding with Liprin-β or CASK unlikely to explain the phase separation and active zone assembly phenotypes we observe.

For thoroughness, we have imaged HSN synapse formation in *hlb-1Δ* (Liprin-beta) and *lin-2Δ* (CASK) backgrounds and find no significant phenotypes:

Left) HSN synapse formation phenotypes of Liprin-β/hlb-1 and CASK/lin-2. Scale bar, 5 µm. Right) Quantification of HSN synapse intensities. The lin-2(n105) temperature sensitive allele was placed at 25˚C for 12 hours before imaging.

Minor points

1. To comprehensively present the potential effects of phosphorylation, it would be beneficial to indicate the previously characterized binding surfaces in Fig. 3C.

Liprin-alpha and SYD-2 interactors are extensive and depicting these without detracting from the identified phosphosites on our figures would be a challenge. We are also hesitant to depict interactors that have not been validated in SYD-2 (most have been identified only in Liprin-α). We have added additional citations that lay out these proteins’ extensive interactors for the interested reader.

2. Could the author add the phosphomimetic mutant SAM(3E) into the Nter condensate? This experiment will further validate whether phosphorylation regulates the interaction between SAM and Nter.

We thank the reviewer for this suggestion. We have performed these experiments and find SAM(3E) partially restores Nter condensate liquidity *in vitro* (Figure 7D), indicating a loss of inhibitory effect and further supporting the proposed model.

3. Exploring whether mammalian liprin-alpha proteins undergo phase separation triggered by the phosphorylation of the three serines would be valuable to establish the broader relevance of their findings. To test the phosphorylation-dependent phase separation, the authors could consider overexpressing liprin-alpha and its phosphomimetic mutant in mammalian cells.

We consider this an interesting proposal as well. These sites are conserved in the mammalian Liprin-αs and phosphorylation has been observed at them in proteome-wide datasets. However, we would like to suggest that these experiments are beyond the scope of the current manuscript.

Reviewer #3: In a landmark paper published in 2020, these authors reported that scaffold proteins at the active zone undergo liquid-liquid phase separation during synapse development in vivo, a process that is further critical for incorporating additional components into the active zone. Here, they provide compelling evidence that SAD-1, a kinase known to regulate synapse formation in different organisms, phosphorylates the active zone protein SYD-2/liprin. This phosphorylation disrupts an intramolecular interaction within SYD-2, triggering LLPS. Based on their experimental data, they propose that SAD-1 is positioned upstream of the active zone formation process and is itself located at nascent synapses directly or indirectly through adhesion molecules. This model is based on convincing data, and the work is of high technical quality, combining biochemistry, genome engineering and high -resolution microscopy in vivo, which is likely unachievable in any other organism. I only have few comments:

- I am puzzled by the extreme variability in fluorescence values throughout this study. For instance, in Fig 2G, the standard deviation of mScar-SYD-2 % intensity values under the control condition is 48, with extreme values from 9 to 229. What could be the biological significance of such fluctuations? It is mentioned in the Materials and Methods section that the images are derived from "synchronized early L4" animals. Could this possibly correspond to an early phase of synaptic maturation, which might explain why even minor developmental shifts could have a strong impact? Providing a kinetics analysis of the accumulation of the different markers (SYD-2, UNC-10, RAB-3), at least in the wild type, would provide insights to rule out the possibility that various genetic conditions and mutations merely postpone or expedite synapse formation.

Throughout the study we have used synchronization methods to capture a precise “L4” stage in development for imaging. We use a synchronization procedure (https://dx.doi.org/10.17504/protocols.io.2bzgap6) and determine an exact developmental stage for imaging based on vulval morphology, which is highly stereotyped and independent of neuronal development. None of the mutants imaged here had alterations in gross development or generation time. These methods ensure a precise developmental time point is imaged in all mutant backgrounds.

Considering these stringent methods, and the fact that all the imaging has been performed with endogenous fluorescent tags, the variance in fluorescence intensities simply reflects a noisy biological reality. Similar variability was seen in our previous study (McDonald et al. 2020). Synapse formation is well stereotyped in time in *C. elegans*, but we have found considerable variability in protein accumulation from animal to animal.

As requested, we have provided some developmental imaging of the kinetics of HSN synapse formation in a new Figure S1. These data show that synaptic components accumulate steadily at the HSN synaptic region from L3 to L4 to adult stages.

We can further be sure that the phenotypes are not due to developmental timing shifts because SYD-2 accumulation has remained normal in the key mutants (*sad-1Δ*, SYD-2(3A)). Thus, the initial steps of synapse formation including SYD-2 recruitment are proceeding as normal, but SAD-1 phosphorylation is required to activate SYD-2 phase separation to build the complete synapse.

- the dependence of SAD-1 on SYG-1, but not SYD-2, for its synaptic localization is an important result. Yet, as mentioned by the authors, these results are inconsistent with those previously published in Patel 2006. The explanation provided attributes this disparity to the prior utilization of overexpressed reporters. It would be interesting to analyze the distribution of SAD-1 in syd-1 mutants to test if SAD-1 remains dependent of SYD-1 for its localization, or if this was also an artefact.

We thank this reviewer for the suggestion. We have quantified the endogenous SAD-1 synaptic localization in a *syd-1Δ* mutant and find a ~30% decrease in SAD-1 localization (Figure 8A-B), similar to the *syd-2Δ* phenotype.

These findings are broadly in agreement with Patel et al. (2006), where we found exogenous SAD-1 localization to be decreased in *syd-2Δ* and *syd-1Δ*. However, with endogenous tags we can now quantitatively see ~70% of SAD-1 localization does not depend on *syd-1* and *syd-2*, leading us to conclude the strength of the prior results may be due to the use of overexpressed transgenes.

In a *syg-1Δ* mutant, SAD-1 loses all synaptic localization (Figure 8A-B). Therefore, the data support synaptic cell adhesion molecule localization of SAD-1, with a minor role for *syd-1* and *syd-2*.

- from the initial SYG-1 paper (2003), SYG-1 concentration peaked at presumptive synaptic sites several hours before the detection of synaptic vesicles. Again, it would be important to observe the kinetics of SYG-1 and SAD-1 localization during HSN synapse formation. Along these lines, do the authors speculate whether SAD-1 is constitutively active or if it requires local activation?

With endogenous fluorescent labels of SYG-1, synaptic vesicles, and active zone components, we observe a steady accumulation of all synaptic components in HSN from L3/L4 molt to adult stages (Figure S1), equivalent to the timepoints imaged in Shen et al. (2003). The divergence from kinetics in the previous study likely reflects transgenic expression from non-native promoters and the lower sensitivity microscopy used at the time.

We do not currently have the tools to test if SAD-1 is specifically activated at presynaptic sites or is constitutively active. SAD-1 localization at synaptic sites may be sufficient for spatial control of SYD-2 activation if the kinase is constitutively active.
